# Insights into the morphology‐productivity relationship of filamentous fungi through small‐scale cultivation and automated microscopy of *Thermothelomyces thermophilus*

**DOI:** 10.1002/btpr.3528

**Published:** 2025-01-23

**Authors:** Katja Rohr, Bertram Geinitz, Johannes Seiffarth, Aydin Anbarani, Sören Bernauer, Matthias Moch, Julia Tenhaef, Wolfgang Wiechert, Katharina Nöh, Marco Oldiges

**Affiliations:** ^1^ Institute of Bio‐ and Geosciences, IBG‐1: Biotechnology Forschungszentrum Jülich GmbH Jülich Germany; ^2^ Institute of Biotechnology RWTH Aachen University Aachen Germany; ^3^ Computational Systems Biotechnology (AVT.CSB) RWTH Aachen University Aachen Germany; ^4^ BASF SE, Nutrition and Health Ludwigshafen am Rhein Germany

**Keywords:** automated microscopy, fed batch, filamentous fungi, morphology‐productivity relationship, phytase, small‐scale cultivation, *Thermothelomyces thermophilus*

## Abstract

Filamentous fungi are a cornerstone in the biotechnological production of enzymes, proteins, and organic acids. However, challenges in understanding and controlling the relationship between morphology and productivity can limit their application. This study addresses these challenges using *Thermothelomyces thermophilus*, a promising thermophilic fungus known for the production of thermostable enzymes. We investigated the effects of environmental conditions on fungal morphology and enzyme production using a combination of microbioreactor cultivation, automated liquid handling, and automated microscopy. Specifically, batch and fed batch cultivations were performed at different pH levels and glucose feeding rates to study their effects on secretory phytase production, fungal growth, and morphology. Results from batch cultivations revealed a two‐fold higher phytase activity at pH 5.5 compared to pH 6.5, with notably smaller fungal fragments at the end of cultivation. Conversely, fed batch cultivations at a feeding rate of 1 g (l h)^−1^ glucose showed a 1.6‐fold higher enzyme activity at pH 5.5, accompanied by much larger fungal aggregates throughout the feeding phase. These findings suggest that large aggregates are associated with high productivity; however, their breakdown further enhances enzyme release, increasing activity in the supernatant. This study not only provides insights on the morphology‐productivity relationship of *T. thermophilus*, but also demonstrates the efficacy of integrating microbioreactors with automated microscopy. This methodology represents a significant advance in the field of fungal biotechnology, paving the way for more efficient industrial bioprocesses.

**Abbreviations**
DO: dissolved oxygen
FP: FlowerPlate
FTU: phytase unit: amount of enzyme that releases 1 μmol of ortho‐phosphate from sodium phytate per minute at pH 5.5 and 37°C
MBR: microbioreactor
STY: space–time yield
*d*_0_
: shaking diameter
*n*
: rotational frequency
*n*_bio_
: number of biological replicates
*n*_tec_
: number of technical replicates
O_2_
: oxygen content of the inlet air
*t*
: time
*T*
: temperature
*V*_L_
: filling volume of cultivation wells
*V*_W_
: total volume of cultivation wells

## 1 INTRODUCTION

Filamentous fungi are a cornerstone in the biotechnological production of enzymes, proteins, and organic acids.^1^ They have been industrially used for more than a century due to distinct advantages, such as remarkable enzyme production and secretion capabilities.^2^, ^3^ Alongside long‐established systems such as *Aspergillus niger* and *Trichoderma reesei*, *Thermothelomyces thermophilus* has emerged as a particularly promising organism.^4^, ^5^ This thermophilic fungus is especially known for its ability to produce an array of thermostable enzymes such as phytases, cellulases, or laccases.^6^, ^7^ In the feed sector, for example, phytases have proven indispensable for improving nutrient availability by releasing bound phosphorus from plant feed materials.^8^

Despite often surpassing bacterial systems in production and secretion capabilities, exploiting filamentous fungi is challenging due to their complex and dynamic morphology.^9^ Their morphology can range from freely dispersed mycelium, which is a branching network of thread‐like structures, to pellets, which are compact, spherical masses of hyphae.^10^ The prevalent morphology of these microorganisms is often closely linked to their productivity and thus has a significant impact on bioprocess productivity.^11^, ^12^, ^13^ Consequently, understanding and eventually controlling the morphology is crucial for optimizing their industrial applications. The interaction between environmental conditions and fungal morphology forms a complex system that requires a methodical, high‐throughput approach to study how varying conditions affect both fungal morphology and product formation.^14^

Over the past decade, small‐scale cultivation devices such as the Ambr (Sartorius, Göttingen, DE), BioLector (Beckman Coulter, Brea, US), or bioREACTOR (2mag AG, München, DE) have gained popularity for methodical, high‐throughput applications.^15^ These microbioreactor (MBR) technologies allow the systematic variation of environmental conditions, facilitating detailed studies of their influence on bioprocesses.^15^ While these systems are well‐established for bacteria, there is limited literature on their use for filamentous fungi. Some first application studies were conducted using the BioLector^16^, ^17^, ^18^ and the bioREACTOR 48.^19^ But these methods are not yet widely used for filamentous fungi, especially in combination with morphological studies. In parallel with these MBR developments, advances in the field of automated microscopy have enabled detailed examinations of morphology.^20^ However, these applications hold severe limitations, such as the use of solid media or difficulties in achieving full automation and high‐throughput.^21^, ^22^, ^23^

A significant step forward was the combination of a BioLector MBR with a robotic liquid handling platform for automated sampling and automated microscopy in the work of Jansen et al. (2021). The authors used the combined system for morphological analysis of pellet‐forming *Aspergillus carbonarius*.^18^ The present study applies this previously established workflow, consisting of MBR cultivation, automated liquid handling, and automated microscopy, to the industrially relevant filamentous microorganism *T. thermophilus*. This fungus is known for its microfilamentous morphology and production of phytase.^6^, ^7^ The techniques used in this study include online measurement of scattered light, dissolved oxygen (DO) and pH of a large number of biological replicates over the time course of cultivation, automated at‐line microscopy with a high number of microscopy images per biological replicate and automated offline measurement of phytase activity. This contrasts with conventional screening methods, which are often based on determining endpoint product concentrations and using manual microscopy.^24^ Specifically, this study is carried out in two steps: (i) Batch cultivation of *T. thermophilus* at two pH‐setpoints, pH 5.5 and pH 6.5, to assess the effect of different pH conditions on phytase activity and fungal growth over the course of cultivation. (ii) Fed batch cultivation of *T. thermophilus*, using the respective pH‐setpoints (pH 5.5 and 6.5) in combination with two different glucose feeding rates, 1 and 2 g (l h)^−1^, to assess their combined effects on phytase activity and fungal growth over the course of cultivation. The significance of this research lies in its potential to explore the critical morphology‐productivity relationship of *T. thermophilus* under environmental conditions similar to those of industrial processes, including pH control and fed batch mode. Such knowledge could pave the way for more efficient bioprocesses in industry, offering substantial contributions to the field of biotechnology.

## 2 MATERIALS AND METHODS

### 2.1 Cultivation media

The following stocks were individually prepared and sterilized: salts pH 5.5 (300 g l^−1^ NaNO_3_, 26 g l^−1^ KCl, 76 g l^−1^ KH_2_PO_4_), glucose (50% (m m^−1^) glucose), casamino acids (100 g l^−1^ casamino acids), magnesium sulfate (246.48 g l^−1^ MgSO_4_ 7 H_2_O), trace elements pH 6.0 (50 g l^−1^ EDTA, 20.05 g l^−1^ ZnSO_4_ 7 H_2_O, 3.92 g l^−1^ MnSO_4_ H_2_O, 4.56 g l^−1^ FeSO_4_ 7 H_2_O, 1.46 g l^−1^ CuSO_4_ 5 H_2_O, 1.37 g l^−1^ Na_2_MoO_4_ 2 H_2_O), antibiotics (20 g l^−1^ penicillin, 50 g l^−1^ streptomycin), uridine (244.20 g l^−1^ uridine), biotin (0.06 g l^−1^ biotin), MES pH 6.0 (213.25 g l^−1^ MES H_2_O). The glucose stock was sterilized by autoclaving at 121°C for 20 min. All other stocks were sterile filtrated using a 0.2 μm polyethersulfone membrane.

Agar plates pH 6.5 were prepared using a basal medium (16 g l^−1^ agar, 230 g l^−1^ saccharose, 5 g l^−1^ yeast extract, 1.12 g l^−1^ uracil), which was completed after autoclaving at 121°C for 20 min with the addition of the following volumes of stocks: 20 mL l^−1^ salts, 20 mL l^−1^ glucose, 10 mL l^−1^ casamino acids, 2 mL l^−1^ magnesium sulfate, 1 mL l^−1^ trace elements, 1 mL l^−1^ antibiotics, 10 mL l^−1^ uridine.

Pre‐culture medium consisted of a basal medium (4.66 g l^−1^ (NH_4_)_2_SO_4_, 0.49 g l^−1^ ${\text{MgSO}}_{4}\cdot 7$ H_2_O, 1.22 g l^−1^ K_2_SO_4_, 0.47 g l^−1^ CaSO_4_ 2 H_2_O, 1.76 g l^−1^ KH_2_PO_4_), which was completed after autoclaving at 121°C for 20 min with the addition of the following volumes of stocks: 20 g l^−1^ glucose, 10 mL l^−1^ casamino acids, 1 mL l^−1^ trace elements, 1 mL l^−1^ antibiotics, 0.1 mL l^−1^ biotin, 100 g l^−1^ MES.

Main culture medium was prepared from a basal medium (6.99 g l^−1^ (NH_4_)_2_SO_4_, 0.49 g l^−1^ MgSO_4_ 7 H_2_O, 1.22 g l^−1^ K_2_SO_4_, 0.47 g l^−1^ CaSO_4_ 2 H_2_O, 1.76 g l^−1^ KH_2_PO_4_), to which the following volumes of stocks were added after autoclaving at 121°C for 20 min: 1 mL l^−1^ trace elements, 1 mL l^−1^ antibiotics, and 0.1 mL l^−1^ biotin. Glucose stock was added as indicated in Section 2.4.

### 2.2 Strain and strain maintenance

The utilized strain *T. thermophilus* was kindly provided by BASF SE (Ludwigshafen am Rhein, DE). Cryo aliquots were obtained by cultivation in a 2 L Erlenmeyer flask with a filling volume of 175 mL, which was incubated for 72 h at 37°C, with 80% humidity, 900 rpm shaking at a 3 mm diameter, and 30% inoculum from a pre‐existing cryo. After cultivation, the cell suspension was cooled on ice water and 10% glycerin was added. It was then split into 1 mL aliquots and stored as cryo spore suspension at −80°C until further use.

### 2.3 Seed train

For each cultivation experiment, 100 μL cryo spore suspension was spread on an agar plate (see Section 1) and incubated for 7 days at 37°C until a loose white layer was visible. Then, each well of a 96‐well square deep well plate was filled with 1 mL of pre‐culture medium (see Section 2.1). Using a pipette tip with a 1.5 mm orifice, a piece of overgrown agar was punched out and added to each well. The plate was sealed with a gas permeable sealing foil and incubated for 72 h at 37°C, with 80% humidity and 900 rpm shaking at a 3 mm diameter. After incubation, the contents of all wells were harvested and combined. For inoculation of the main culture, 70% of main culture medium (see Section 2.1) and 30% of the harvested pre‐culture were combined.

### 2.4 Microbioreactor cultivation

MBR cultivations were performed in a BioLector Pro (Beckman Coulter, Brea, US) using a microfluidic FlowerPlate (FP) (M2P‐MTP‐MF32C‐BOH1). During cultivation, scattered light as a measure of the biomass concentration, DO and pH were measured non‐invasively with the built in optodes. The measurement interval was set to 10 min. Cultivation conditions were 37°C, 3 mm shaking diameter, ≥ 85% humidity, 0.8 mL initial well filling volume, and 1400 rpm rotational frequency. The pH‐setpoints were pH 5.5 or 6.5, as indicated in the figures, and the pH was adjusted with 3 M KOH. The plates were sealed with a gas‐permeable sealing foil (M2P‐F‐RSMF32‐1). In batch experiments, 20 g l^−1^ glucose was used as the sole carbon source and 35% oxygen was used in the inlet air. In fed batch experiments, 5 g l^−1^ glucose was used in the batch phase and 15 g l^−1^ glucose (based on the starting volume) was additionally fed. In the batch phase, 21% oxygen was used in the inlet air, which was increased to 35% oxygen during the fed batch phase.

### 2.5 Automated sampling and sample processing

The BioLector Pro is integrated into a Freedom EVO 200 liquid handling platform (Tecan, Männedorf, CH). The liquid handling platform sampled the BioLector cultures automatically at predetermined times, whereby one complete well was harvested per sample. A portion of the samples used for microscopy was automatically diluted 5‐70‐fold with 0.9% NaCl based on the cultivation time at the time of sampling. After dilution, the samples were stored at 4°C until further use.

### 2.6 Automated microscopy

Automated microscopy was performed as described by Jansen et al.,^18^ using a custom‐built injection station and a flow chamber mounted under the microscope. In contrast to Jansen et al.,^18^ 800 μL of sample was injected using a continuous flow at a velocity of 3 μL s^−1^. A microscope chamber with a height of 250 μm and a width of 5 mm (ibidi, GrÃ¤felfing, DE) and a microscopic magnification of 150 were used in the Eclipse Ti2 inverted light microscope (Nikon Europe B.V., Amstelveen, NL).

### 2.7 Phytase activity measurement and space–time yield calculation

After cultivation, the cell suspension was filtered through a 0.2 μm polyethersulfone membrane and the permeate was diluted in acetate buffer pH 4.5 (5.85 g l^−1^ CH_3_COONa 3 H_2_O, 3.24 mL l^−1^ acetic acid 17.6 M), if needed. The volumetric phytase activity was then determined using the vanadate‐molybdate method.^25^ The activity was measured in phytase units (FTUs), which is the amount of enzyme that releases 1 μmol of ortho‐phosphate from sodium phytate per minute at pH 5.5 and 37°C. For the phytase activity assay, 180 μL phytic acid solution pH 4.5 (4.83 g l^−1^ phytic acid sodium salt hydrate dissolved in acetate buffer) was mixed with 20 μL processed sample and incubated at 30°C. Samples of 30 μL were taken at 3, 6, 9, and 12 min intervals, mixed with 90 μL coloring solution (0.39 g l^−1^ NH_4_VO_3_, 16.67 g l^−1^ (NH_4_)_2_MoO_4_ 3 H_2_O, 1.97 mL l^−1^ 25% ammonium hydroxide, 119.25 mL l^−1^ 65% nitric acid) and incubated for 15 min at room temperature before photometric analysis at 415 nm. The volumetric activity was deduced using phytase standards prepared from lyophilized enzyme stocks (0–25 FTU ml^−1^). The absorption of standards at 415 nm was plotted over time (3, 6, 9, and 12 min) and a linear regression was performed. The slope of the standards was then plotted against their concentration to create a calibration curve.

The space–time yield (STY) was calculated by dividing the difference in activity at two points by the time difference between the same time points, according to Equation (1).

(1) $$\text{space}‐\text{time yield}\left[\mathrm{FTU}\;{\left(\mathrm{ml}\;h\right)}^{-1}\right]=\frac{{\text{activity}}_{t_{2}}\left[\mathrm{FTU}\;{\mathrm{ml}}^{-1}\right]-{\text{activity}}_{t_{1}}\left[\mathrm{FTU}\;{\mathrm{ml}}^{-1}\right]}{t_{2}\left[h\right]-t_{1}\left[h\right]}.$$

### 2.8 Data processing and display

Data analysis and visualization were conducted using Python 3.9.7, bletl 1.3.1,^26^, ^27^ Matplotlib 3.5.1,^28^, ^29^ Numpy 1.21.5,^30^ Pandas 1.4.1,^31^, ^32^ and Seaborn 0.11.2.^33^, ^34^ Cultivation plots show the mean and standard deviation for the replicates of each condition as a continuous line and a colored area around the continuous line, respectively. Measurements from sampled wells were excluded from figures and calculations after sampling. This exclusion is necessary as empty wells no longer provide a usable signal. Before plotting, the lowest scattered light value from all replicates of one condition at measurement cycle 3 was used as a blank value. The scattered light measurements of each replicate were compared to this baseline and the difference was subtracted from the values. Scattered light was additionally adjusted for dilution due to pH control or feeding by multiplying with the ratio of current to initial well volume. Phytase activity measurements were volume‐corrected accordingly.

## 3 RESULTS AND DISCUSSION

### 3.1 Batch cultivations

**FIGURE 1:**
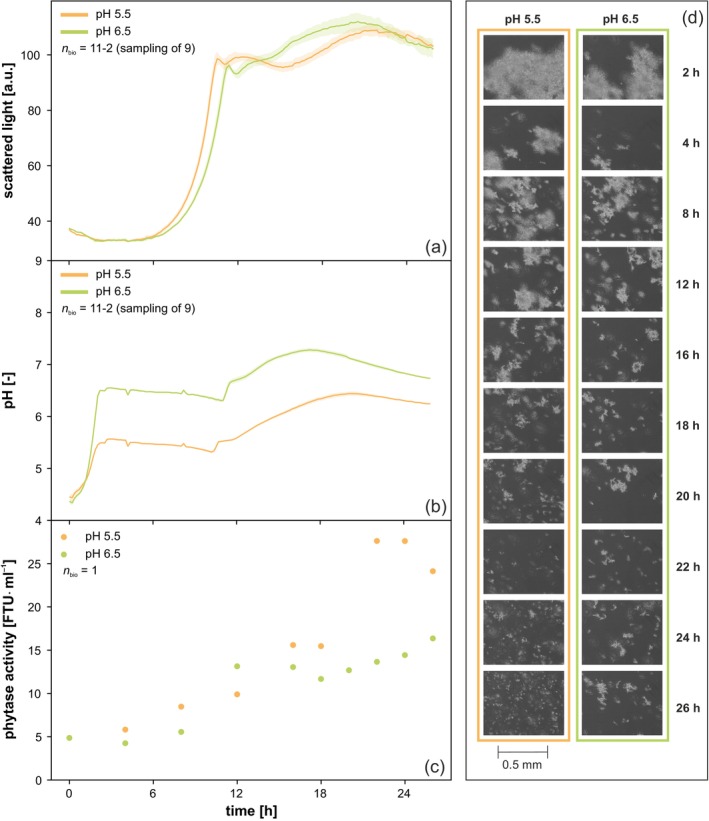
Batch cultivation of *Thermothelomyces thermophilus* with microfluidic pH control. (a) Scattered light, (b) pH and (c) phytase activity over time. (d) Exemplary microscopic images taken fully automatically during cultivation. See Figure S1 for an enlarged version of the microscopic images. Cultivation conditions: *T. thermophilus*, microfluidic FP, *n* = 1400 rpm, d_0_ = 3 mm, *V*_W_ = 3.2 mL, *V*_L_ = 0.8 mL, humidity ≥ 85%, O_2_ = 35%, *T* = 37°C, 20g l^−1^ glucose, *n*_bio_ = 11 with sampling of 9.

To establish a workflow that provides insights into the morphology‐productivity relationship of *T. thermophilus*, the first step was setting up a batch cultivation. With the aim of providing conditions that mimic those found in industrial settings, this cultivation was conducted using pH control (3 M KOH). During cultivation, the impact of two different pH‐setpoints, pH 5.5 and 6.5, on fungal growth and phytase activity were assessed. These pH‐setpoints were chosen as they lie within the optimal growth range of *T. thermophilus* (pH 4.5 to 7.0) and are representative of conditions found in industrial applications.^35^ The results are presented in Figure 1.

The online measurement of scattered light, an indicator of fungal growth, initially showed a similar progression for cultures at both pH‐setpoints (Figure 1a). However, over the course of cultivation, the fungi cultivated at pH 5.5 showed slightly faster growth than those at pH 6.5, as indicated by reaching the initial maximum of scattered light approx. 0.8 h earlier. This faster growth at pH 5.5 was also evident by an earlier increase in pH, also by approx. 0.8 h at around 10 h process time (Figure 1b). An increase in pH, as observed in these cultivations, is typically found after the depletion of glucose when only one‐sided pH control is applied. One‐sided pH control with 3 M KOH had been deliberately chosen for the batch mode to match the fed batch conditions. In fed batch mode, the BioLector Pro, which is capable of feeding two solutions simultaneously, allows the application of a (glucose) feed in combination with one‐sided pH control.

Phytase activity was similar for both pH conditions during the first 18 h of cultivation (Figure 1c). However, towards the end of cultivation, from 22 h onwards, up to two‐fold higher enzyme activity was observed at pH 5.5. At the end of cultivation at 26 h, cultures at pH 5.5 had a 1.5‐fold higher phytase activity than those at pH 6.5. These results clearly demonstrate that the applied pH‐setpoints are well‐suited to achieve productivity variations in *T. thermophilus*.

Morphology analysis is based on the qualitative assessment of 15 different microscopy images per sampling point and condition. It revealed the presence of very large and medium‐sized structures in both conditions during the first 8 h of cultivation (Figure 1d). A notable morphological change occurred between 8 and 12 h, marked by the breakdown of larger structures into predominantly medium‐sized aggregates. This shift coincided with the depletion of the primary carbon source, as indicated by a peak in the scattered light signal and the increase in pH. It is therefore very likely that the observed breakdown of larger structures was caused by glucose depletion.

After glucose depletion, a further increase in scattered light was observed (Figure 1a). This increase is unlikely to reflect an increase in biomass, but rather an optical effect resulting from morphological changes. Specifically, the observed breakdown of larger fungal aggregates into smaller fragments during this phase increases the number of particles in suspension, which enhances light scattering. These results highlight the importance of integrating morphological analysis with scattered light measurements to accurately interpret fungal growth dynamics.

From 22 h onwards, there was a clear morphological difference between the cultures. At pH 5.5, significantly smaller fragments were present, which we did not find at pH 6.5. This morphological variation coincided with the up to two‐fold higher phytase activities at pH 5.5. It is widely recognized that the morphology of filamentous fungi has a strong influence on their productivity.^11^, ^12^ The results of this cultivation run thus suggest that smaller fragments of *T. thermophilus* may lead to higher productivity and therefore phytase activity under the given batch conditions. Alternatively, larger aggregates may exhibit significant productivity but secrete only a portion of the produced enzyme. The breakdown of theses aggregates at pH 5.5 around 22 h could then release intracellularly accumulated phytase, resulting in the observed increase in enzyme activity in the supernatant. It should be noted that despite the differences in morphology, the progression of scattered light at this time was very comparable between the two pHs. This underlines the importance of using several complementary measurement techniques for screening and process development with filamentous fungi, as such important relationships may otherwise go undetected.

### 3.2 Bridging laboratory screening and industrial production: Applying fed batch conditions

**FIGURE 2:**
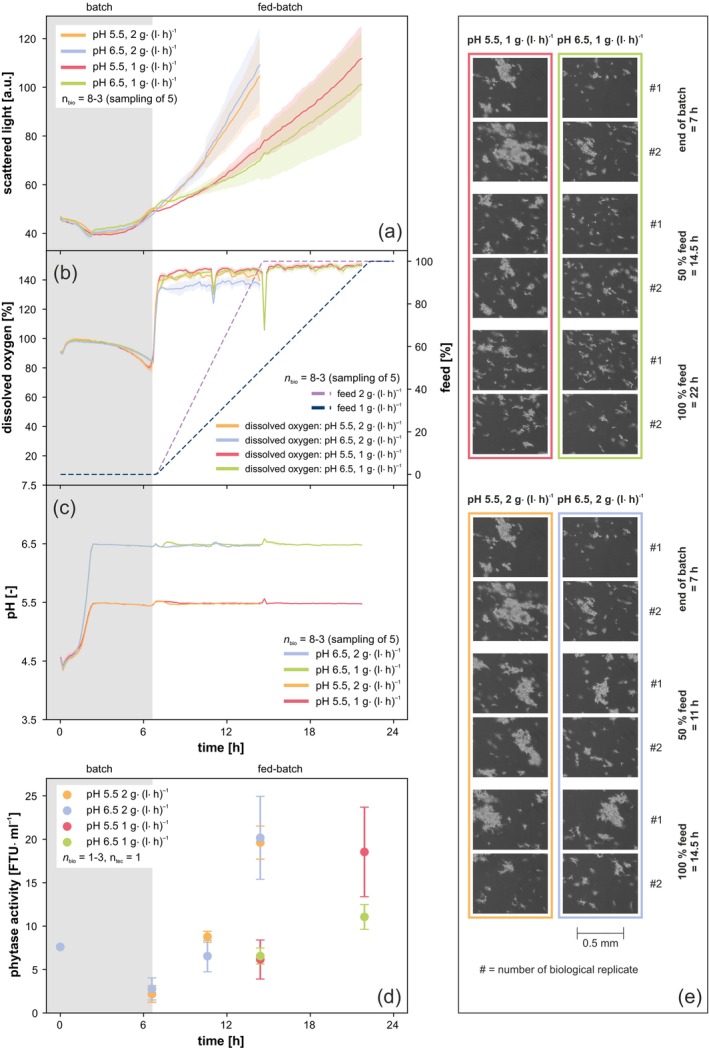
Fed batch cultivation of *Thermothelomyces thermophilus* with microfluidic pH control. (a) Scattered light, (b) DO and feed, (c) pH and (d) phytase activity over time. (e) Exemplary microscopic images taken fully automatically during cultivation. See Figure S2 for an enlarged version of the microscopic images. Cultivation conditions: *T. thermophilus*, microfluidic FP, *n* = 1400 rpm, *d*_0_ = 3 mm, *V*_W_ = 3.2 mL, *V*_L_ = 0.8 mL, humidity ≥ 85%, *T* = 37°C. Batch: 5g l^−1^ glucose, O_2_ = 21%, *n*_bio_ = 8 with sampling of 2; fed batch: 15g l^−1^ glucose as a constant feed with a rate of 1 or 2 g (l h)^−1^, O_2_ = 35%, *n*_bio_ = 8 with sampling of 5.

After establishment of the batch cultivation protocols with two different pH‐setpoints (pH 5.5 and 6.5), the next step was to apply a microfluidic fed batch to even more closely mimic industrial conditions. During cultivation, the effect of the previously tested pH‐setpoints in combination with two feeding rates on fungal growth, phytase activity, and morphology was assessed. In the present gene cassette, phytase production is regulated by the glaA promoter derived from *A. niger*. While Ganzlin and Rinas^36^ report that glucose acts as an inducer rather than a repressor of the glaA promoter in *A. niger*, our observations from previous batch cultivations at pH 5.5 revealed a more than two‐fold increase in phytase activity after depletion of the primary carbon source until the end of cultivation (Figure 1c). Consequently, we have tailored the fed batch process to provide glucose for cell growth in the batch phase and to facilitate phytase production in the fed batch phase by avoiding the drawbacks associated with a potential carbon catabolite repression. Thus, the glucose feeding rates, 1 and 2 g (l h)^−1^, were chosen to target carbon limitation. These rates were determined based on glucose consumption rates from previous experiments (data not shown). The results of the fed batch cultivations are presented in Figure 2.

Scattered light at the end of the batch phase showed a steeper increase for cultures at pH 5.5 compared to pH 6.5, indicating faster growth (Figure 2a). This observation is additionally supported by the earlier increase of DO, indicating an earlier carbon depletion (Figure 2b). These results are consistent with observations from the previous batch experiment (Figure 1a). During the fed batch phase, cultures fed at 2 g (l h)^−1^ had a very similar progression of scattered light for both pHs. In contrast, cultures fed at 1 g (l h)^−1^ showed a slightly faster increase of scattered light at pH 5.5 than cultures with the same feeding rate at pH 6.5. Comparing the two feeding rates, the faster feeding rate, 2 g (l h)^−1^, resulted in faster growth at both pHs, which is the expected result in a carbon‐limited fed batch cultivation. Nevertheless, similar final scattered light values were reached for all conditions, ranging only within 10 a.u. of each other. In contrast to the batch cultivations, the pH‐setpoints 5.5 and 6.5 were very well‐matched throughout the cultivation, even though one‐sided pH control with 3 M KOH was applied (compare Figures 1b and 2c). This is most likely an effect of the switch to fed batch cultivation.

Samples for phytase measurements were taken at the beginning and end of batch and after the addition of 50% and 100% feed volume. As the same total amount of glucose was fed at both feeding rates, end point samples were taken at 15 and 22 h respectively. At the end of cultivation, very similar phytase activities were detected for cultures at pH 5.5 and 6.5, which were both fed with 2 g (l h)^−1^ (Figure 2d yellow and blue). Their final activities were 20 ± 2 and 20 ± 5 FTU ml^−1^ (both *n*_bio_ = 3, *n*_tec_ = 1). In contrast to these results, a clear difference in phytase activity was observed between cultures at pH 5.5 and 6.5, which were both fed with 1 g (l h)^−1^ (Figure 2d red and green). With final activities of 18 ± 5 and 11 ± 1 FTU ml^−1^ (both *n*_bio_ = 3, *n*_tec_ = 1), the activities at pH 5.5 were 1.6 times higher than those at pH 6.5. Yet, compared to the faster feeding rate of 2 g (l h)^−1^, the activity at pH 5.5 was very similar. A direct comparison of the final phytase activities is given in Table 1.

**TABLE 1:** Comparison of key performance indicators for fed batch cultivation of *T. thermophilus* with microfluidic pH control. Phytase activity given is the activity at the end of cultivation. Space–time yield was calculated for the fed batch phase according to Equation (1). All data was obtained with *n*_bio_ = 3 and *n*_tec_ = 1.

Condition	Activity	Space–time yield
	[FTU ml^−1^]	[FTU (ml h)^−1^]
pH 5.5, 1g (l h)^−1^	18 ± 5	1.1
pH 6.5, 1g (l h) ^−1^	11 ± 1	0.5
pH 5.5, 2g (l h) ^−1^	20 ± 2	2.2
pH 6.5, 2g (l h) ^−1^	20 ± 5	2.2

It is crucial to not only consider the total enzyme activity, but also the STY. The STY in the fed batch phase was calculated by dividing the difference in activity between the end of feed and the end of batch by the time elapsed between these two points (Equation 1). It was 1.1 FTU (ml h)^−1^ for cultures at pH 5.5 fed with 1 g (l h)^−1^, but 2.2 FTU (ml h)^−1^ for all cultures that were fed with 2 g (l h)^−1^. This represents a two‐fold improvement in this important key performance indicator. Moreover, the STY of cultures at pH 6.5 fed with 1 g (l h)^−1^ was only 0.5 FTU (ml h)^−1^. Compared to these results, the adjustment of pH and/or feeding rate achieves a two‐fold and four‐fold increase in the STY. A direct comparison of the STYs is given in Table 1. These results clearly demonstrate that the workflow is well‐suited to induce a variation in productivity through the applied pH and feed conditions.

Morphology analysis is based on the qualitative assessment of 100 images per sampled biological replicate. Two biological replicates were sampled at the end of batch for each condition, while three were sampled at each sampling point during the feeding phase, resulting in a total of 200–300 different images per sampling point and condition. In cultures fed with 2 g (l h)^−1^, medium to large aggregates were present after the addition of 50% and 100% feed, irrespective of pH (Figure 2e yellow and blue). Conversely, no large aggregates were present in cultures fed with 1 g (l h)^−1^. However, medium‐sized aggregates were observed at pH 5.5, whereas only small pieces were present at pH 6.5 (Figure 2e red and green). The cause of these differences between the two feeding rates is likely the stronger carbon limitation of the slower feeding rate. As can be seen in Figure 2b, no decrease in DO is observed during feeding. Thus, both feeds represent C‐limited conditions. It is therefore clear that the slower feeding rate results in higher stress and a stronger carbon limitation for the cells than the faster feeding rate. This reduced availability of glucose could lead to the breakdown of larger fungal aggregates, analogous to the observations in the batch experiments after carbon depletion (Figure 1d). The more pronounced carbon limitation of the slower feeding rate also offers an explanation for the differences observed in morphology and phytase activity between the cultures at pH 5.5 and those at pH 6.5 (Figure 2d,e red and green). In this particular Case, at a feeding rate of 1 g (l h)^−1^, pH 6.5 led to a morphology of predominantly small fragments and a 1.6‐fold lower phytase activity, while no similar effect of pH on morphology and phytase activity was observed at a feeding rate of 2 g (l h)^−1^. Our hypothesis is that the cells are more susceptible to the effects of pH on morphology and productivity due to the higher environmental stress.

Based on these observations, the appearance of small fungal fragments in a fed batch cultivation of *T. thermophilus* may indicate suboptimal environmental conditions, such as an insufficient feeding rate and suboptimal pH, which can result in lower product formation. As product analysis in cultivation processes is usually performed offline, the presented at‐line morphology analysis could be used in the running process to adjust the conditions, for example, to increase the current feeding rate, in order to improve product formation and thus STY.

### 3.3 Evaluating workflow insights: A side‐by‐side comparison of batch and fed batch results

A comparative analysis of batch and fed batch cultivation results yields several key insights. In batch experiments, similar morphologies and phytase activities were observed between pH 5.5 and 6.5, as long as glucose was present. After depletion of the primary carbon source, a breakdown of aggregates into small fragments was observed at pH 5.5. Simultaneously, phytase activity continued to increase more than two‐fold until the end of cultivation. Based on these results, two hypotheses were put forward. (i) Small fungal fragments of *T. thermophilus* lead to high productivity. (ii) Large aggregates yield high productivity, but the produced enzyme is only partly secreted into the cultivation medium. However, the breakdown of larger aggregates effectively releases previously produced phytase, increasing enzyme activity in the supernatant.

In fed batch experiments, small fungal fragments combined with low phytase activities were observed at pH 6.5 and a feeding rate of 1 g (l h). These results clearly contradict the first hypothesis that small fungal fragments lead to high productivity. Since larger fungal aggregates and a 1.6‐ to 1.8‐fold higher phytase activity were observed in all other conditions, the second hypothesis is likely to apply. However, no breakdown of the aggregates was detected. Therefore the second part of this hypothesis could neither be rejected nor confirmed.

Even after extensive analysis, the similar morphology at different pH values between batch and fed batch cultivations cannot be conclusively explained. It remains unclear, why a breakdown of large aggregates into small fragments was observed in batch cultivations at pH 5.5, whereas exclusively small fragments were observed during the feeding period of fed batch experiments at pH 6.5 (1 g (l h)^−1^). Obviously, batch cultivations and carbon‐limited fed batch cultivations provide fundamentally different environmental conditions. This could be a factor in the similar morphology at different pH values, however further investigations are required. Nevertheless, these results clearly show that our workflow is well‐suited to apply different environmental conditions to a large number of biological replicates in order to induce changes in productivity and morphology of *T. thermophilus*. The insights gained demonstrate the significant value of our workflow in investigating the important morphology‐productivity relationship.

## 4 CONCLUSIONS

We have successfully adapted and applied the workflow previously developed by Jansen et al. (2021), consisting of MBR cultivation, automated sampling and automated microscopy, to the industrially relevant phytase producer *T. thermophilus*. In batch cultivations, we found a two‐fold higher phytase activity at pH 5.5 compared to pH 6.5, accompanied by the presence of significantly smaller fungal fragments at the end of cultivation. Similarly, in fed batch cultivations with a feeding rate of 1 g (l h)^−1^, a 1.6‐fold higher phytase activity was detected at pH 5.5 compared to pH 6.5. Interestingly, this higher enzymatic activity was accompanied by comparatively much larger fungal aggregates throughout the entire feeding phase. These observations led us to the hypothesis that large aggregates yield high productivity. The potential breakdown of these aggregates effectively releases previously produced phytase, further increasing enzyme activity in the supernatant. These results clearly demonstrate that the workflow and the conditions applied, including pH control and fed batch mode, are well‐suited to induce changes in productivity and morphology of *T. thermophilus*. To the authors' knowledge, this has enabled the first holistic study of the complex relationship between environmental conditions, morphology, and productivity of this industrially relevant filamentous fungus. This understanding is pivotal for designing and optimizing industrial bioprocesses.

Future research should focus on distinguishing between intracellular and secreted enzyme activity to better understand the relationship between fungal morphology and productivity. For example, quantification of intracellular phytase levels relative to extracellular secretion under varying conditions could validate the hypothesis of enzyme release during aggregate breakdown. In addition, while this study has developed methods to perform comprehensive morphological studies through automated sampling and microscopy imaging, a new bottleneck has emerged in terms of automated image analysis and subsequent morphological characterization. In particular, real‐time analysis of fungal morphology at the production line promises to provide important insights into ongoing biotechnological processes. However, this step requires fast and reliable automated image processing. While powerful image analysis methods based on deep learning have been developed,^37^ they need to be adapted for the analysis of fungal morphologies in our flow chamber setup and trained on annotated data sets.^38^ Here, our automated high‐throughput microscopy represents a critical step forward in efficiently acquiring these image datasets and linking AI‐based morphology characterizations to productivity metrics. This advancement will pave the way for the design of more efficient industrial bioprocesses, thereby offering substantial contributions to the field of biotechnology.

## AUTHOR CONTRIBUTIONS

**K. R**. led the scientific work, including planning, execution and analysis of all experiments, as well as writing the manuscript; **B. G**. helped to interpret the data and plan the experiments, was responsible for project administration/supervision and substantially revised the manuscript; **J. S**. and **K. N**. helped with morphological image analysis; **A. A**. and **S. B**. assisted in the interpretation of the data, planning of experiments and provided the strain and media protocols; **M. M**. assisted in conducting the experiments; **J. T**. made a significant contribution to the establishment of the product assays; **W. W**. helped to finalize the manuscript; **M. O**. was responsible for funding acquisition, project administration/supervision and revised the manuscript.

## FUNDING INFORMATION

Funding was received from the “Enabling Spaces” program “Helmholtz Innovation Labs” of the German Helmholtz Association to support the “Microbial Bioprocess Lab—A Helmholtz Innovation Lab”. Funding was furthermore received from BASF SE.

## CONFLICT OF INTEREST STATEMENT

The authors declare no conflicts of interest.

## Supporting information


**FIGURE S1:** Exemplary microscopic images taken fully automatically during batch cultivation of *Thermothelomyces thermophilus*. Cultures at (a) pH 5.5 and (b) pH 6.5. Cultivation conditions: *T. thermophilus*, microfluidic FP, *n* = 1400 rpm, *d*_0_ = 3 mm, *V*_W_ = 3.2 mL, *V*_L_ = 0.8 mL, humidity ≥ 85%, *O*_2_ = 35%, *T* = 37°C, 20 g l^−1^ glucose, *n*_bio_ = 11 with sampling of 9.

**FIGURE S2:** Exemplary microscopic images taken fully automatically during fed batch cultivation of *T. thermophilus*. Cultures at (a) pH 5.5 and (b) pH 6.5, both fed with 1 g (l h)^−1^ glucose. Cultures at (c) pH 5.5 and (D) pH 6.5, both fed with 2 g (l h)^−1^ glucose. Cultivation conditions: *T. thermophilus*, microfluidic FP, *n* = 1400 rpm, *d*_0_ = 3 mm, *V*_W_ = 3.2 mL, *V*_L_ = 0.8 mL, humidity ≥ 85%, *T* = 37°C. Batch: 5 g l^−1^ glucose, O_2_ = 21%, *n*_bio_ = 8 with sampling of 2; fed batch: 15 g l^−1^ glucose as a constant feed with a rate of 1 or 2 g (l h) ^−1^, O_2_ = 35%, *n*_bio_ = 8 with sampling of 5.

## Data Availability

The data that support the findings of this study are available from the corresponding author upon reasonable request.
