# Supplementary figures and images for: Insights into the morphology‐productivity relationship of filamentous fungi through small‐scale cultivation and automated microscopy of Thermothelomyces thermophilus
Source: Biotechnol Prog. 2025 Jan 23;41(3):e3528. doi: 10.1002/btpr.3528 (PMC12171336; doi:10.1002/btpr.3528)

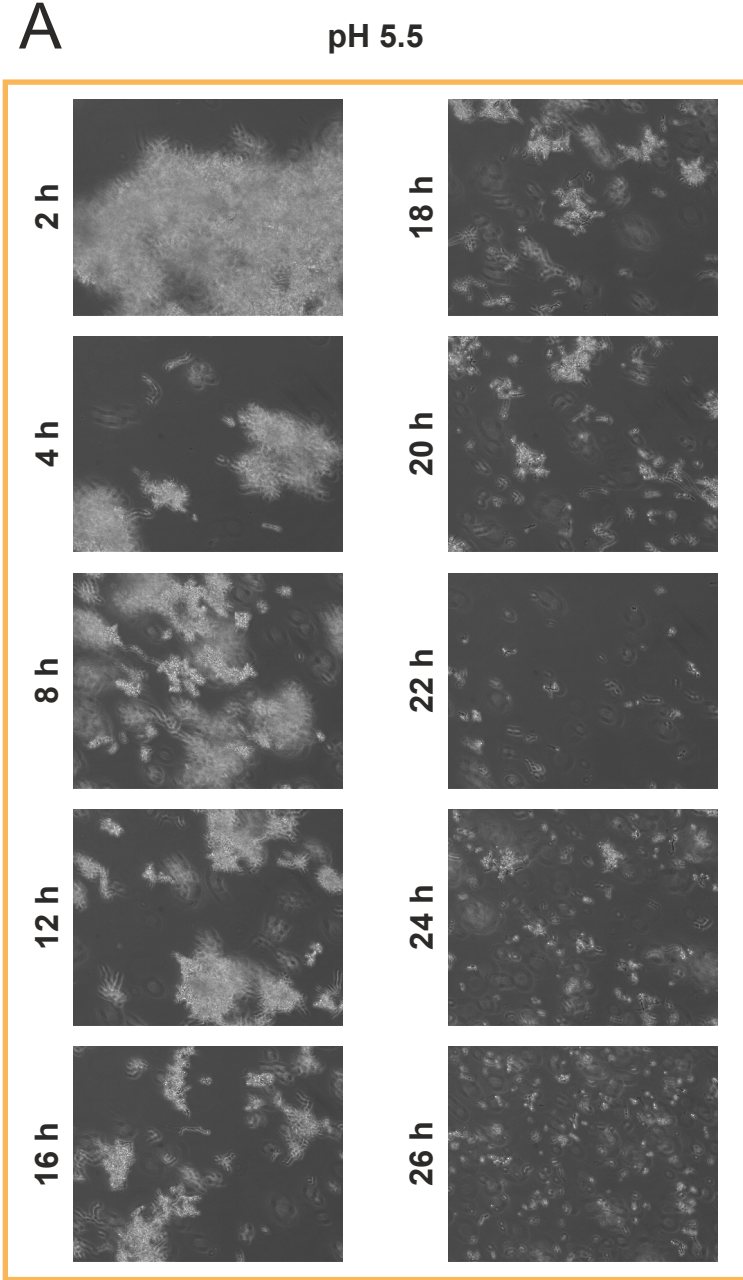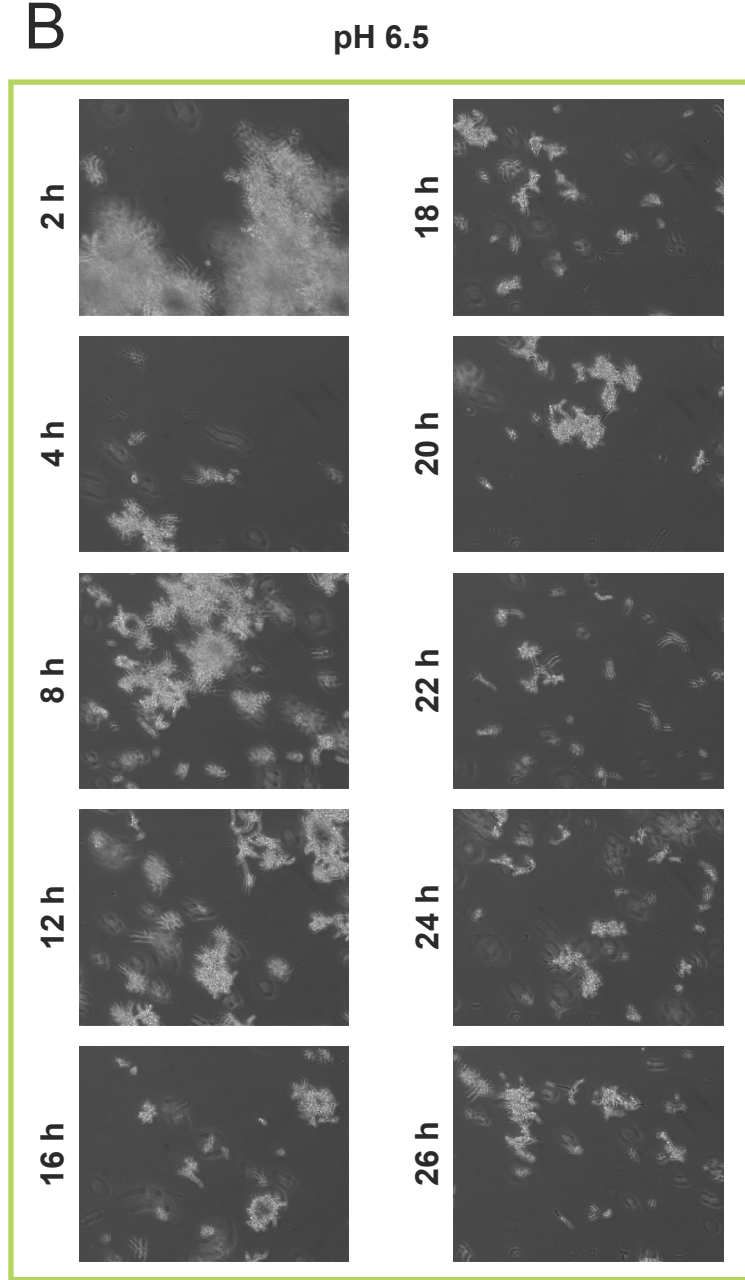

Supplement: Supplementary file 1 — FIGURE S1: Exemplary microscopic images taken fully automatically during batch cultivation of Thermothelomyces thermophilus. Cultures at (a) pH 5.5 and (b) pH 6.5. Cultivation conditions: T. thermophilus, microfluidic FP, n = 1400 rpm, d 0 = 3 mm, V W = 3.2 mL, V L = 0.8 mL, humidity ≥ 85%, O 2 = 35%, T = 37°C, 20 g l−1 glucose, n bio = 11 with sampling of 9. [file BTPR-41-e3528-s002.pdf]
