# Supplementary material for: Insights into the morphology‐productivity relationship of filamentous fungi through small‐scale cultivation and automated microscopy of Thermothelomyces thermophilus
Source: Biotechnol Prog. 2025 Jan 23;41(3):e3528. doi: 10.1002/btpr.3528 (PMC12171336; doi:10.1002/btpr.3528)
Supplement: Supplementary file 2 — FIGURE S2: Exemplary microscopic images taken fully automatically during fed batch cultivation of T. thermophilus. Cultures at (a) pH 5.5 and (b) pH 6.5, both fed with 1 g (l h)−1 glucose. Cultures at (c) pH 5.5 and (D) pH 6.5, both fed with 2 g (l h)−1 glucose. Cultivation conditions: T. thermophilus, microfluidic FP, n = 1400 rpm, d 0 = 3 mm, V W = 3.2 mL, V L = 0.8 mL, humidity ≥ 85%, T = 37°C. Batch: 5 g l−1 glucose, O2 = 21%, n bio = 8 with sampling of 2; fed batch: 15 g l−1 glucose as a constant feed with a rate of 1 or 2 g (l h) −1, O2 = 35%, n bio = 8 with sampling of 5. [file BTPR-41-e3528-s001.pdf]

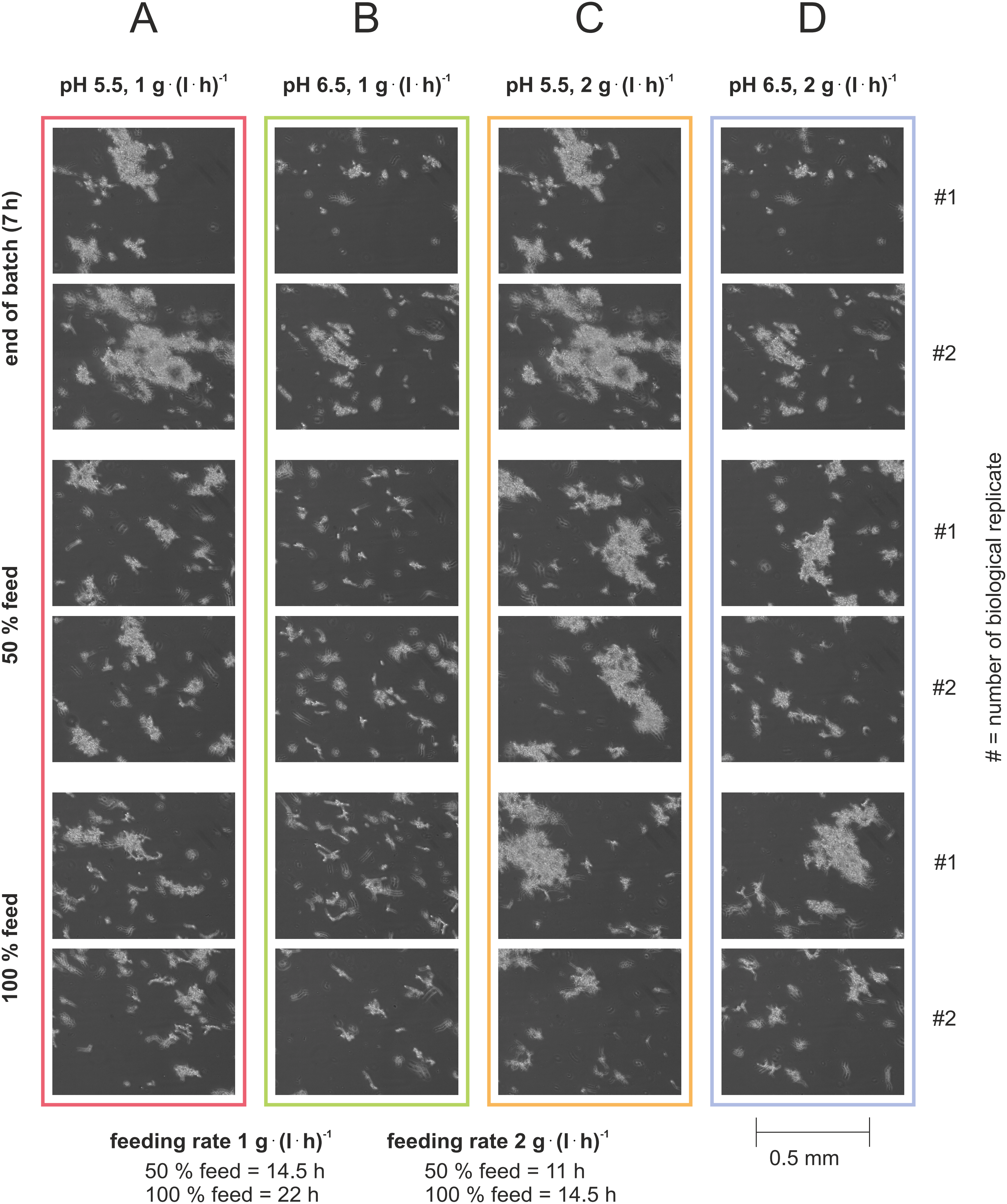
A
B
C
D
pH 5.5, 1 g · (l · h)^-1
pH 6.5, 1 g · (l · h)^-1
pH 5.5, 2 g · (l · h)^-1
pH 6.5, 2 g · (l · h)^-1
end of batch (7 h)
50 % feed
100 % feed
#1
#2
#1
#2
#1
#2
# = number of biological replicate
feeding rate 1 g · (l · h)^-1
50 % feed = 14.5 h
100 % feed = 22 h
feeding rate 2 g · (l · h)^-1
50 % feed = 11 h
100 % feed = 14.5 h
0.5 mm
